# Neurobiological influences on event perception: the role of catecholamines

**DOI:** 10.1093/ijnp/pyaf008

**Published:** 2025-02-21

**Authors:** Foroogh Ghorbani, Xianzhen Zhou, Veit Roessner, Bernhard Hommel, Astrid Prochnow, Christian Beste

**Affiliations:** Cognitive Neurophysiology, Department of Child and Adolescent Psychiatry, Faculty of Medicine, TU Dresden, Dresden 01307, Germany; Cognitive Neurophysiology, Department of Child and Adolescent Psychiatry, Faculty of Medicine, TU Dresden, Dresden 01307, Germany; Cognitive Neurophysiology, Department of Child and Adolescent Psychiatry, Faculty of Medicine, TU Dresden, Dresden 01307, Germany; German Center for Child and Adolescent Health (DZKJ), Partner Site Leipzig/Dresden, Dresden 01307, Germany; Cognitive Neurophysiology, Department of Child and Adolescent Psychiatry, Faculty of Medicine, TU Dresden, Dresden 01307, Germany; School of Psychology, Shandong Normal University, Jinan 250014, Shandong Province, China; Cognitive Neurophysiology, Department of Child and Adolescent Psychiatry, Faculty of Medicine, TU Dresden, Dresden 01307, Germany; Cognitive Neurophysiology, Department of Child and Adolescent Psychiatry, Faculty of Medicine, TU Dresden, Dresden 01307, Germany; German Center for Child and Adolescent Health (DZKJ), Partner Site Leipzig/Dresden, Dresden 01307, Germany; School of Psychology, Shandong Normal University, Jinan 250014, Shandong Province, China

**Keywords:** catecholamines, event perception, event segmentation, methylphenidate

## Abstract

**Background:**

Event segmentation, the cognitive process of parsing continuous experiences into discrete events, plays a fundamental role in how humans perceive and interact with their environment. Guided by Event Segmentation Theory, this study investigates the modulation of event segmentation by the catecholaminergic system by methylphenidate (MPH).

**Methods:**

Healthy adult participants (*N* = 52) engaged in a double-blind, counter-balanced, placebo-controlled experiment in which they watched a movie and identified event boundaries under placebo and MPH conditions.

**Results:**

With the same information given, MPH increased the likelihood that the information was considered meaningful. Crucially, the number of situational changes and participant’s prior experience had an interactive effect on the probability of event segmentation. There was a stronger relationship between environmental information and segmentation probability when catecholaminergic levels were elevated by MPH in addition to previous experience.

**Conclusions:**

The catecholaminergic system modulates how incoming information is segmented to build meaningful episodes. Prior experience supports the effects of MPH to unfold. These findings underscore the complex interplay between neurochemical modulation and cognitive processes involved in event perception.

**Significance Statement**

This study investigates how our brain divides continuous experiences into meaningful events, a process known as event segmentation. We investigated how neurotransmitters of the catecholaminergic system influence this process. Administering methylphenidate, we show that the ability to detect event boundaries improved when catecholamine neurotransmitter levels were elevated, but only when participants had previous experience with the task. Our results suggest that the catecholaminergic system plays a crucial role in how we organize our experiences.

## INTRODUCTION

In our daily lives, we are continuously exposed to a stream of information (Barker, 1963). One of the fundamental cognitive processes for understanding this incoming information is event segmentation, which is explained by the Event Segmentation Theory (EST) (Zacks et al., 2007; Zacks, 2020). EST addresses our understanding of the world by breaking down continuous experiences into meaningful units, referred to as event segments (Zacks et al., 2007, 2009; Zacks, 2020; Sargent et al., 2013). According to EST, a cognitive working event model held in working memory actively represents the current situation. This model is generated based on environmental information (bottom-up processing), the perception of which is in turn biased by the current working event model, and on inputs from semantic memory in a top-down fashion (known as event schemata), containing previously acquired information about the sequential structure of activities (Zacks et al., 2007). The current working event model forecasts near-future states of the world (Zacks et al., 2007), which are compared to actual occurrences by an error detection system. When a prediction fails, the current working event model is terminated and reconstructed to reflect the new experience or situation (Zacks et al., 2007; Hard et al., 2011). This transition marks an event boundary, that is, the end of one event segment and the beginning of another one.

Error detection and working memory are both central to the event segmentation process (Zacks et al., 2007; Zacks, 2020), and influenced by the catecholaminergic system, including dopamine and norepinephrine (Holroyd and Coles, 2002; Zacks et al., 2007; Robbins and Arnsten, 2009; Cools and D’Esposito, 2011; Caetano et al., 2012; Zacks, 2020). Therefore, it is plausible that event segmentation is affected by catecholamines. An involvement of brain regions associated with the catecholaminergic system in event segmentation has already been demonstrated (Zacks and Sargent, 2010). More specifically, post error adjustment, such as updating of the deficient working event model, and the stability of working memory representations, such as the working event model, are influenced by the catecholaminergic system (Holroyd and Coles, 2002; Caetano et al., 2012; Mückschel et al., 2020a). Intriguingly, previous experience can mitigate or even reverse the effects of catecholamines on adaptive behaviors (Bensmann et al., 2019; Mückschel et al., 2020a, 2020b). Thus, prior experiences and familiarity with a specific situation, as represented in event schemata, likely interact with the effects of the catecholaminergic system on event segmentation.

An experimental manipulation of the catecholaminergic system can be achieved through the administration of methylphenidate (MPH), a dual dopamine and norepinephrine transporter blocker known to increase postsynaptic catecholamine levels (Volkow et al., 1999; Skirrow et al., 2015; Faraone, 2018). MPH increases “gain control” by enhancing the signal-to-noise ratio in neural information processing (Servan-Schreiber et al., 1990; Li et al., 2001; Yousif et al., 2016; Ziegler et al., 2016; Adelhöfer et al., 2018; Gao et al., 2024; Koyun et al., 2024a, 2024b) and thus facilitating response control and selection (Aston-Jones and Cohen, 2005a). Increased gain control reflects an amplification of the responsiveness of the information processing system to incoming signals (Salinas and Thier, 2000). In the context of EST, this suggests that incoming sensory information may influence the likelihood of segmentation behavior by modulating the working event model and error detection processes, potentially increasing or decreasing the probability of setting an event boundary, depending on the interaction between catecholaminergic modulation and the cognitive processes involved in event segmentation.

The current study aims to investigate the effects of catecholaminergic modulation on event segmentation and to explore how this modulation interacts with information stored in event schemata. To this end, healthy adult participants underwent 2 sessions (either administered MPH or placebo) in a randomized, double-blind, counter-balanced, placebo-controlled design. During these sessions, they were asked to identify meaningful events within a movie. As the influence of the catecholaminergic system on cognitive functions appears to be modulated by prior task experience (Bensmann et al., 2018; Bensmann et al., 2019; Mückschel et al., 2020b; Eggert et al., 2021), we hypothesize a similar interaction in the context of event segmentation: performance is assumed to be impaired when catecholaminergic levels are elevated in conjunction with previous experience (ie, event schemata). This research uniquely aims to elucidate how prior knowledge and the regulation of the catecholaminergic system influence our perception of the world and our organization of it into coherent and meaningful events.

## METHOD

### Participants

*N* = 73 healthy participants aged between 20 and 30 years (mean age 24.4 ± 2.6 years; 34 females) participated in this study. *N* = 6 participants had to be excluded due to problems in the data collection process (eg, dropout, randomization errors leading to the same substance being administered at both appointments), and *N* = 4 were excluded as they did not meet the inclusion criteria through the screening phase (see below). During the data analysis, *N* = 3 individuals were excluded for having too few responses for data analyses in one of their appointments, and N = 8 individuals were excluded as one of their appointments was identified as an outlier regarding their number of responses (i.e., participants whose total number of responses was 3 SD below and/ or above the average were excluded). The final sample thus consisted of *N* = 52 participants (mean age 24.4 ± 2.9 years; 24 females). All participants were right-handed, had normal or corrected-to-normal vision, and reported neither neurological or psychiatric conditions nor regular drug and/or medication intake. The inclusion criteria were ensured in a brief screening during the recruitment procedure and by a detailed questionnaire that covered not only the medical history but also asked for substance abuse issues of the participants. Subclinical symptoms were not assessed, as they were considered part of the expected variability among individuals without psychiatric diagnoses. Participants were recruited through the University Clinic Carl Gustav Carus’ and the Technical University of Dresden’s database as well as advertisements. At the time of the experiment, participants provided written informed consent and were compensated for their participation after the second session. The local ethics committee of the Medical Faculty of the Technical University of Dresden approved the study. No part of the study procedures and analyses have been preregistered.

### MPH Administration

The study was conducted using a randomized, double-blind, counter-balanced, placebo-controlled design, where each subject participated in 2 sessions, scheduled approximately 7 days apart. Participants received MPH in one of the sessions and a placebo compound in the other, the order of which was double-blind and counter-balanced across subjects. This resulted in *N* = 25 subjects of the final sample having received the placebo compound in the first and MPH in the second session, and *N* = 27 subjects of the final sample having received MPH in the first and the placebo compound in the second session. The individual MPH dosage was individually calculated based on the participant’s body weight (0.25 mg/kg) in line with previous studies (Bensmann et al., 2019; Mückschel et al., 2020b; Prochnow et al., 2024a). Since MPH plasma levels peak 1 to 3 hours after oral administration (Challman and Lipsky, 2000; Rösler et al., 2009), the experimental procedures were started approximately 75 minutes after MPH administration.

### Task

Participants were asked to perform an event segmentation task, in which they were presented with a movie and were asked to press the space key whenever they perceived that something in the movie was about to end and something new was about to start. Participants were further informed that there are no right or wrong answers and that their pure individual assessment is important for the study. In order to practice this task and to clarify potential questions, participants were presented with a video clip in which a man assembled a boat using a “Duplo” construction block (Zacks et al., 2009). After the practice phase, participants watched the movie “The Red Balloon” (Le ballon rouge, 1956) in the context of an event segmentation task. Several specific characteristics of the movie make it suitable for examining the event segmentation, such as a low amount of spoken language, frequent situational changes, and a chronological storyline (Zacks et al., 2009, 2010; Magliano and Zacks, 2011). The movie was divided into 4 7 to 10-minute clips (lengths of 463.3, 468.4, 446.2, and 600.6 seconds) (Zacks et al., 2009), with breaks between each episode in which it was possible to resume the task by tapping the space button. The videos were shown to the participants and their responses were recorded using “Presentation” (Neurobehavioral Systems Inc.). In each session of the experiment, participants watched the movie once.

The movie “The Red Balloon” has been scored frame by frame with respect to the occurrence of situational changes (eg, changes in the location of agents or the interaction of characters) by Zacks et al. (2009) and this scoring has already been applied in several studies (Zacks et al., 2009, 2010; Kurby et al., 2014; Ghorbani et al., 2024; Prochnow et al., 2024b, 2024c). Therefore, the established rating scheme was utilized in the present study as well. Besides the occurrence of situational changes, the type of the situational change was coded by Zacks et al. (2009). As analyses concerning these types of changes are not the study’s main focus, they are presented in the Supplementary Material.

### Statistical Analyses

**Figure 1.**
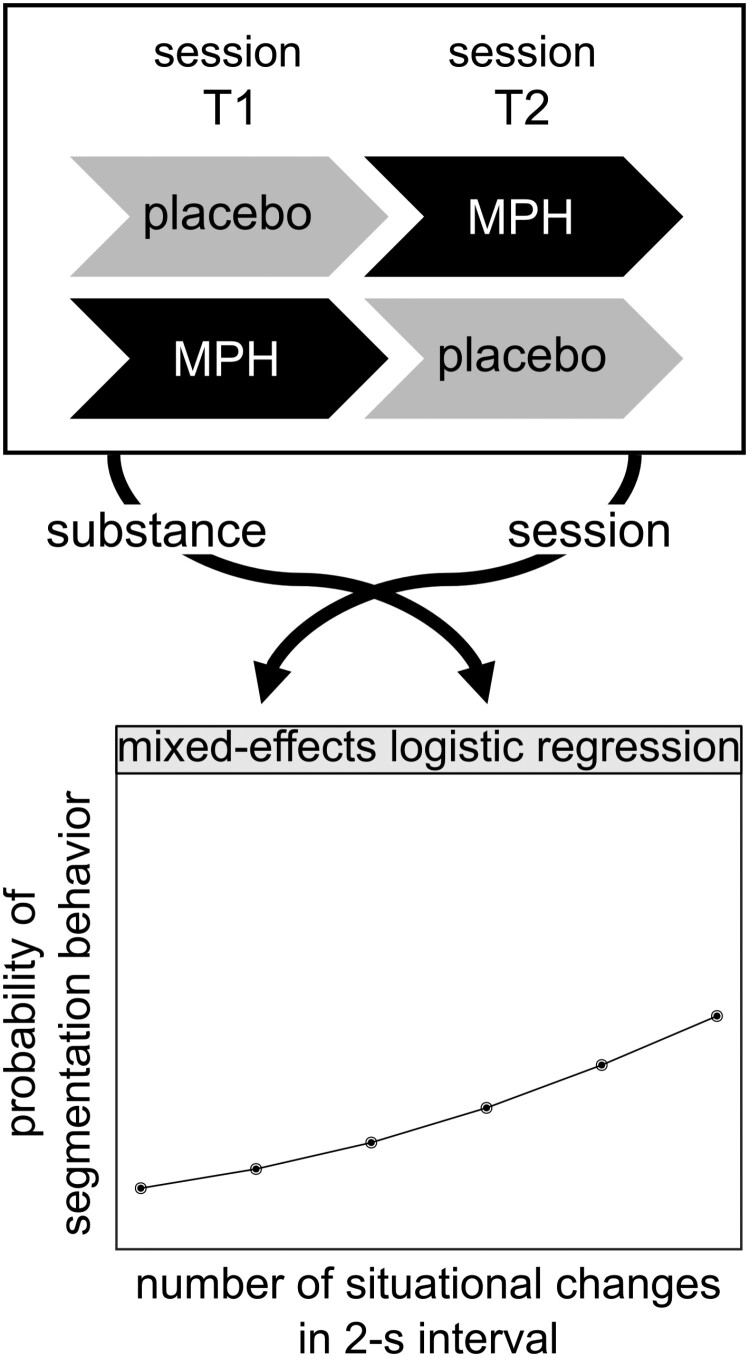
Schematic illustration of the MPH administration design (top) as part of the statistical model. Subjects received either a placebo compound (placebo, gray boxes) in the first session T1 and MPH (MPH, black boxes) in the second session T2, or vice versa. Both session and substance were entered into the mixed-effects logistic regression model as predictors in addition to the predictor number of changes in a 2-second interval. All 3 predictors were used to estimate the probability of the segmentation behavior. MPH, methylphenidate.

To statistically analyze the data, mixed-effects logistic regression (R version 4.3.3, “glmer” function) was performed. For the analysis of the event segmentation behavior during “The Red Balloon” (Le ballon rouge, 1956), the clips of the movie were divided into time bins with a length of 2 s. Then, the number of changes within such a 2-s interval according to the scoring by Zacks et al. (2009) was counted for each interval, resulting in 518 intervals with no changes, 278 intervals with one change, 106 intervals with 2 changes, 52 intervals with 3 changes, 29 intervals with 4 changes, and 4 intervals with 5 changes. Moreover, for each 2-second interval, it was coded if a response by the participant occurred within this interval or not. The regression model was used to examine the influence of the predictors number of changes (0-5), the substance (placebo [0] vs MPH [1]), and the session (T1 [0] vs T2 [1]) on the probability of segmentation behavior (no response [0] vs response [1]). The statistical model including the design of the MPH administration is schematically illustrated in Figure 1.

Furthermore, the influence of the predictor gender of the subject (between-subjects; Male [0] vs Female [1]) was evaluated in an additional analysis.

The random intercept for subjects was estimated to account for the variability between subjects, and odds ratios (ORs) were calculated based on the coefficient results of fixed effect to be able to compare the influence of the different predictors. In the case of significant results, particularly significant interaction effects, the effects will be further examined using post hoc logistic regression models, following a hierarchical testing framework to reduce the risk of Type I errors. The results of the logistic regression models are given with estimated coefficients and standard error along with the z- and *P*-values for the statistical test and the OR with the corresponding 95% confidence interval (95% CI) as effect size. The alpha level for all analyses was set to 05.

## RESULTS

**Figure 2.**
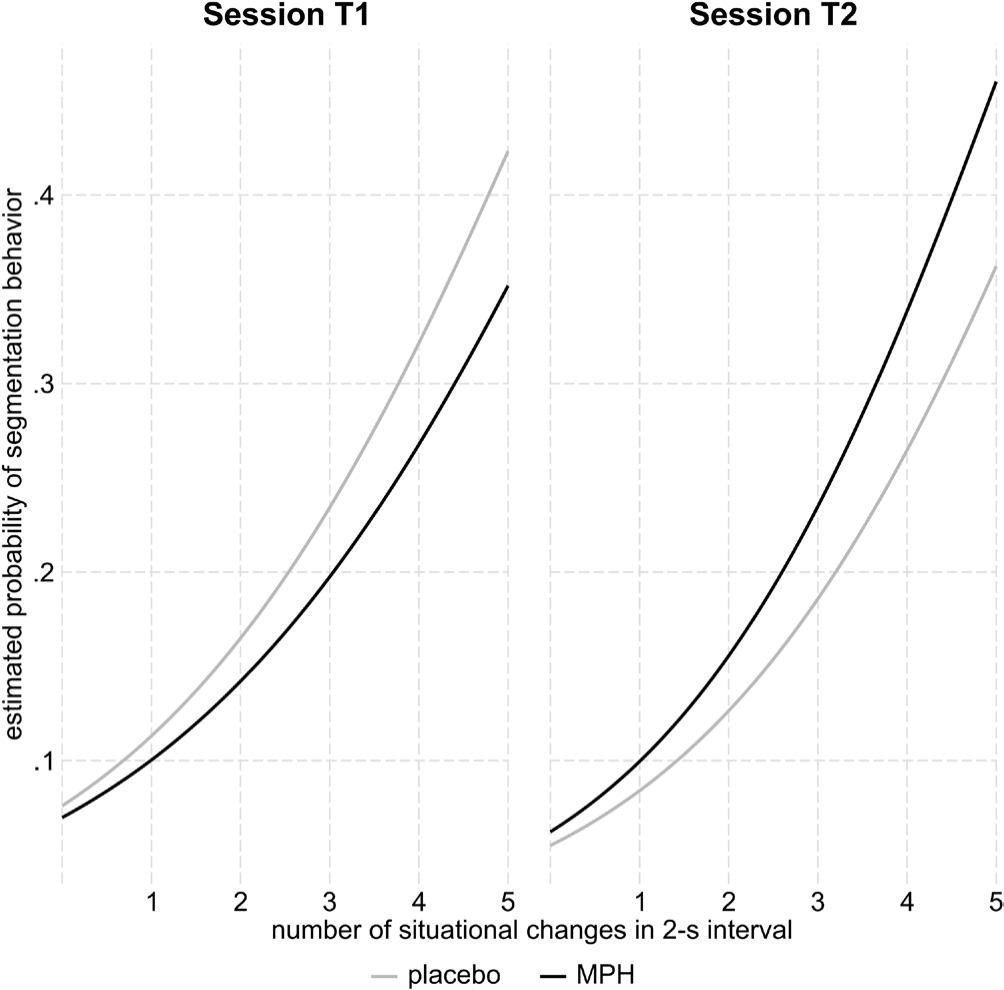
Probability of segmentation behavior (y-axis) as a function of the number of changes in a 2-second interval (x-axis) in sessions T1 (left) and T2 (right). Gray lines indicate placebo, black lines indicate MPH. MPH, methylphenidate.

The mixed-effects logistic regression regarding the probability of segmentation behavior with significant intercept (−2.50 ± .13, z = −19.62, *P* < .001, OR = .08, 95% CI, .06-.11) revealed a significant influence of the predictor number of changes (.44 ± .02, z = 27.35, *P* < .001, OR = 1.55, 95% CI, 1.50-11.60) and a significant interaction of the predictors number of changes, substance, and session (.10 ± .03, z = 2.96, *P* = .003, OR = 1.10, 95% CI, 1.03-1.17). The results are displayed separately for the sessions in Figure 2.

### Post Hoc Analyses Separately for Sessions

Considering only the T1 sessions (intercept: −2.50 ± .13, z = −19.64, *P* < .001, OR = .08, 95% CI, .06-.11), neither the substance (−.09 ± .18, z = −.50, *P* = .616, OR = .92, 95% CI, .65-1.29) nor the interaction of the number of changes and the substance (−.04 ± .02, z = −1.82, *P* = .068, OR = .96, 95% CI, .92-1.00) had a significant influence on the probability of segmentation.

Considering the T2 sessions (intercept: −2.86 ± .14, z = −20.97, *P* < .001, OR = .06, 95% CI, .04-.07), however, there was no sole effect of the substance (.09 ± .20, z = .47, *P* = .642, OR = 1.10, 95% CI, .75-1.61), but an interaction of the number of changes and the substance (.06 ± .02, z = 2.72, *P* = .007, OR = 1.06, 95% CI, 1.02-1.12). The influence of the number of changes on the probability of segmentation was larger under the influence of MPH (.52 ± .02, z = 31.17, *P* < .001, OR = 1.69, 95% CI, 1.63-1.74; intercept: −2.77 ± .15, z = −18.52, *P* < .001, OR = .06, 95% CI, .05-.08) compared to placebo (.46 ± .02, z = 27.27, *P* < .001, OR = 1.58, 95% CI, 1.53-1.63; intercept: –2.86 ± .13, z = −22.23, *P* < .001, OR = .06, 95% CI, .04-.07).

### Post Hoc Analyses Separately for Substances

Considering only the sessions under the influence of placebo (intercept: −2.50 ± .13, z = –18.91, *P* < .001, OR = .08, 95% CI, .06-.11), a significant influence of the session on the segmentation probability was shown (–.36 ± .18, z = –1.98, *P* = .048, OR = .69, 95% CI, .48-1.00). However, the interaction of session and the number of changes was not significant (.02 ± .02, z = .89, *P* = .376, OR = 1.02, 95% CI, .98-1.07).

Considering only the sessions under the influence of MPH (intercept: -2.59 ± .13, z = −19.72, *P* < .001, OR = .08, 95% CI, .06-.10), there was no sole significant effect of the session on segmentation probability (–.18 ± .19, z = –.97, *P* = .331, OR = .83, 95% CI, .57-1.21). Yet, the interaction of the predictors session and number of changes had a significant influence on the segmentation probability (.13 ± .02, z = 5.45, *P* < .001, OR = 1.13, 95% CI, 1.08-1.19). Thereby, the influence of the number of changes on the segmentation probability was larger when MPH was administered in the second session (.52 ± .02, z = 31.17, *P* < .001, OR = 1.69, 95% CI, 1.63-1.74; intercept: –2.77 ± .15, z = –18.52, *P* < .001, OR = .06, 95% CI, .05-.08) compared to when administered in the first session (.39 ± .02, z = 24.68, *P* < .001, OR = 1.49, 95% CI, 1.44-1.53; intercept: –2.59 ± .12, z = -21.80, *P* < .001, OR = .08, 95% CI, .06-.10).

### Effects of Gender

The mixed-effects logistic regression regarding the probability of segmentation behavior with significant intercept (–2.45 ± .17, z = –13.96, *P* < .001, OR = .08, 95% CI, .06-.12) did not reveal any significant influence of the interaction between predictors substance, session, and gender (.11 ± .06, z = 1.79, *P* = .073, OR = 1.12, 95% CI, 0.98-1.27).

## DISCUSSION

In the current study, we aimed to examine the role of the catecholaminergic system in event segmentation according to the EST (Zacks and Swallow, 2007; Zacks, 2020), particularly its interplay with established event schemata from prior experience in a particular setting. To this end, we tested healthy adult participants in an event segmentation task using the movie “The Red Balloon” (Le ballon rouge, 1956) over 2 sessions. In a double-blind, counter-balanced, and randomized design, participants were administered either MPH or a placebo compound. Thus, we could assess both the manipulation of the catecholaminergic system (placebo or MPH) and the amount of previous experience (no previous experience in the first session, and with previous experience in the second session) when examining the influence of situational changes on the probability of event segmentation.

Our results showed that previous experience, the manipulation of the catecholaminergic system, and the number of situational changes had an interactive influence on the probability of event segmentation. While in the first session, the relationship between the number of situational changes and segmentation probability was not affected by catecholaminergic influences, these predictors showed an interactive effect on segmentation probability in the second session. The relationship between the number of situational changes and segmentation probability was increased when MPH was administered compared to the placebo compound. Moreover, MPH increased the influence of situational changes on segmentation probability more in the second session compared to the first. In other words, there was a stronger relationship between environmental information and segmentation probability when catecholaminergic levels were elevated by MPH in addition to previous experience.

Considering these results in the context of EST (Zacks and Swallow, 2007; Zacks, 2020), elevated catecholaminergic levels likely enhance the maintenance of the working event model, which is closely linked to working memory processes (Mückschel et al., 2020a). Additionally, the adjustment to errors, that is, the updating of the working event model might be improved by higher catecholaminergic levels (Holroyd and Coles, 2002; Caetano et al., 2012), leading to stronger working event models and more distinct event boundaries due to better adjustments after prediction errors. Consequently, the event segmentation’s dependence on environmental information should increase, as this information is more rigorously compared against the predictions of the working event model. However, this effect appears only when catecholaminergic levels are elevated in conjunction with prior experience, as an established event schema enables the working event model to form useful predictions, which is not feasible without previous experience, regardless of catecholamine levels.

The observed results may be also explained by gain control mechanisms, where increased gain control strengthens the input-output relationship (Servan-Schreiber et al., 1990; Aston-Jones and Cohen, 2005b). Gain control can be enhanced by both increased catecholaminergic levels (Servan-Schreiber et al., 1990; Aston-Jones and Cohen, 2005b) and learning or previous experience (Dosher and Lu, 1998; Gold et al., 1999; Friedrich and Beste, 2020). In line with this, the current findings suggest that gain control is maximized when previous experience and elevated catecholaminergic levels coincide, showing a stronger relationship between input (amount of situational changes) and output (segmentation probability) only in the second session (Aston-Jones and Cohen, 2005b).

However, previous studies indicated that this relationship between performance and gain control influenced by both previous experience and elevated catecholamine levels by MPH, might be best described by an inverted U-shaped function (Servan-Schreiber et al., 1990; Aston-Jones and Cohen, 2005b; Mückschel et al., 2020a). This suggests that too much gain control might rather impair performance, as the gain control level exceeds the optimum range in the sense of an “overshoot” (Bensmann et al., 2018; Mückschel et al., 2020a; Eggert et al., 2021) which seems contradictory to the current study’s results. A reason for this might be that previous studies employed more “classical” paradigms using a stimulus-response structure (Bensmann et al., 2018; Mückschel et al., 2020a; Eggert et al., 2021), requiring a stronger focus, achievable through increasing catecholaminergic system toward an optimal level of gain control (Ghosh and Maunsell, 2024; Keehn and Kadlaskar, 2023). While in these tasks, an “overshoot” of gain control might diminish focus and thus impair task performance, the current study’s event segmentation paradigm requires a broader focus to capture the entire scene dynamically. Thus, the strength of the focus on details likely varies with catecholamine levels, and differences in focus requirements likely explain the observed discrepancies in performance outcomes.

Combining both theoretical concepts, the current results suggest that an “overshoot” in gain control allows more perceptual information to be processed due to less strict focusing. This additional perceptual information is used in the error detection mechanism as a reference for the validity of the current working event model, enhancing the influence of environmental information on event segmentation behavior. Further, when both more perceptual information and established event schemata are available, the working event model is based on more extensive information. As a result, predictions might be clearer, making prediction errors more likely and thus triggering the setting of event boundaries. Therefore, it might be mainly the amount of perceptual information that is allowed into the system and can be utilized that is influenced by the catecholaminergic system. Furthermore, our findings contribute to a broader understanding of how neurochemical modulation through different neurotransmitters could shape a perceptual and cognitive organization, reflecting shifts in neural processing and integration across different brain states (ie, active perceptual processing and the resting state) (Conio et al., 2020; Northoff and Hirjak, 2024).

In conclusion, our study highlights the intricate interplay between the catecholaminergic system and prior experience in event segmentation. Elevated catecholaminergic levels, in conjunction with established event schemata, enhance the integration of perceptual information, thereby refining the working event model. This improved model facilitates more precise predictions and error detection, leading to clearer event boundaries. Future research should further explore this dynamic to better understand the underlying neural mechanisms and their implications for cognitive processes.

## Supplementary Material

pyaf008_suppl_Supplementary_Materials

## Data Availability

The data underlying this article will be shared on reasonable request to the corresponding author.
