# Supplementary material for: Neurobiological influences on event perception: the role of catecholamines
Source: Int J Neuropsychopharmacol. 2025 Feb 21;28(2):pyaf008. doi: 10.1093/ijnp/pyaf008 (PMC11879076; doi:10.1093/ijnp/pyaf008)
Supplement: pyaf008_suppl_Supplementary_Materials [file pyaf008_suppl_supplementary_materials.docx]

**Supplemental Material**

**Neurobiological Influences on Event Perception: The Role of Catecholamines**

F. Ghorbani, X. Zhou, V. Roessner, B. Hommel, A. Prochnow, C. Beste

**Supplemental Analysis: Types of Situational Changes**

*Materials and Methods*

The situational change coding provided by Zacks et al. (2009) contained information regarding the type of the changes that occurred throughout the movie. The situational changes were categorized in nine different categories:

1. Character changes: difference in the agents in the current frame compared to the previous frame
2. Character-character changes: difference in the way agents interact in the current frame compared to the previous frame
3. Character-object changes: difference in the way an agent uses an object in the current frame compared to the previous frame
4. Temporal changes: discontinuities in time between two consecutive frames
5. Large space changes: difference of the camera view on the scene in the current frame compared to the previous frame
6. Small space changes: difference in the location of an agent in the current frame compared to the previous frame
7. Cause changes: when the actions in the current frame cannot be explained by actions in the previous frame
8. Goal changes: when the goals of an agent change in the current frame compared to the previous frame
9. Scene changes: cuts between two camera settings

The occurrence of each of these types of changes was coded for the 2-s intervals (no occurrence [0] vs occurrence [1]) and was included as predictor in the logistic regression model. To statistically analyze the data, mixed-effects logistic regression (R version 4.3.3, ‘glmer’ function) was performed. The logistic regression model with the probability of segmentation as outcome considered the predictors occurrence of type of change (no occurrence [0] vs occurrence [1]) simultaneously for all nine types of changes, substance (placebo [0] vs MPH [1]) and session (T1 [0] vs T2 [1]). The random intercept for subjects was estimated to account for the variability between subjects, and odds ratios were calculated based on the coefficient results of fixed effect to be able to compare the influence of the different predictors. In the case of significant results, particularly significant interaction effects, the effects will be further examined using post-hoc logistic regression models. The results of the logistic regression models are given with estimated coefficients and standard error along with the z- and p-values for the statistical test and the odds ratio (OR) with the corresponding 95% confidence interval (95% CI) as effect size.

*Results*

The mixed-effects logistic regression model with significant intercept (-2.60 ± .13, z = -19.95, p < .001, OR = .07, 95% CI: .06 - .10) revealed significant effects of the situational change types Character (.74 ± .05, z = 14.11, p < .001, OR = 2.09, 95% CI: 1.88 – 2.31), Character-character (.77 ± .07, z = 11.79, p < .001, OR = 2.17, 95% CI: 1.90 – 2.46), Character-object (.24 ± .08, z = 3.04, p = .002, OR = 1.27, 95% CI: 1.09 – 1.48), Temporal (.24 ± .11, z = 2.25, p = .025, OR = 1.27, 95% CI: 1.03 – 1.57), Large space (.27 ± .08, z = 3.29, p = .001, OR = 1.31, 95% CI: 1.12 – 1.54), Small space (.41 ± .05, z = 7.59, p < .001, OR = 1.51, 95% CI: 1.36 – 1.68), Cause (.27 ± ..07, z = 4.03, p < .001, OR = 1.31, 95% CI: 1.15 – 1.50) and Scene (.24 ± ..07, z = 3.37, p < .001, OR = 1.27, 95% CI: 1.10 – 1.45). However, neither the predictors substance (-.11 ± .18, z = -.60, p = .547, OR = .90, 95% CI: .63 – 1.28), session (-.34 ± .18, z = -1.89, p = .059, OR = .71, 95% CI: .50 – 1.01) nor their interaction (.25 ± .36, z = .71, p = .480, OR = 1.29, 95% CI: .64 – 2.60) had a significant influence on the segmentation probability. Regarding the interactions of the type of changes with the substance and session, there was a significant interaction of substance and session with the occurrence of Character changes (.37 ± .11, z = 3.40, p < .001, OR = 1.44, 95% CI: 1.17 – 1.78) as well as with the occurrence of Large space changes (-.65 ± .17, z = -3.79, p < .001, OR = .52, 95% CI: .37 - .73). The odds ratios of the Character and the Large space changes as a function of substance and session are displayed in Supplemental Fig. 1.

Post-hoc mixed-effects logistic regression models were then calculated separately for each session. Regarding Character changes in the T1 sessions, there was a significant interaction of substance with the occurrence of Character changes (-.20 ± .07, z = -2.75, p = .006, OR = .81, 95% CI: .70 - .94). When the T1 session was under the influence of placebo, the occurrence of Character changes significantly influenced the segmentation probability (.73 ± .05, z = 14.09, p < .001, OR = 2.08, 95% CI: 1.88 – 2.31), which was, however, to a lesser extent, also the case when the T1 session was under the influence of MPH (.53 ± .05, z = 9.93, p < .001, OR = 1.70, 95% CI: 1.53 – 1.88). Regarding Character changes in the T2 sessions, there was a significant interaction of substance with the occurrence of Character changes (.17 ± .08, z = 2.22, p = .027, OR = 1.19, 95% CI: 1.02 – 1.39). When the T2 session was under the influence of placebo (.59 ± .06, z = 10.43, p < .001, OR = 1.80, 95% CI: 1.61 – 2.01), the influence of the occurrence of Character changes was lower than when the T2 session was under the influence of MPH (.76 ± .06, z = 13.83, p < .001, OR = 2.14, 95% CI: 1.92 – 2.39).

Regarding Large space changes in the T1 sessions, there was a significant interaction of substance with the occurrence of Large space changes (.38 ± .12, z = 3.20, p = .001, OR = 1.46, 95% CI: 1.16 – 1.84). When the T1 session was under the influence of placebo, the occurrence of Large space changes significantly influenced the segmentation probability (.27 ± .08, z = 3.29, p = .001, OR = 1.31, 95% CI: 1.12 – 1.54). However, when the T1 session was under the influence of MPH, the influence of the occurrence of Large space changes on the segmentation probability was even larger (.65 ± .08, z = 7.69, p < .001, OR = 1.91, 95% CI: 1.62 – 2.26). Regarding Large space changes in the T2 sessions, there was a significant interaction of substance with the occurrence of Large Space changes (-.27 ± .13, z = -2.18, p = .030, OR = .76, 95% CI: .60 – .97). The occurrence of Large space changes only had a significant influence on the segmentation probability when the T2 session was under the influence of placebo (.34 ± .09, z = 3.98, p < .001, OR = 1.42, 95% CI: 1.19 – 1.68). However, when the T2 session was under the influence of MPH, Large space changes did not have a significant influence on the segmentation probability (.08 ± .09, z = .87, p = .387, OR = 1.08, 95% CI: .91 – 1.29).


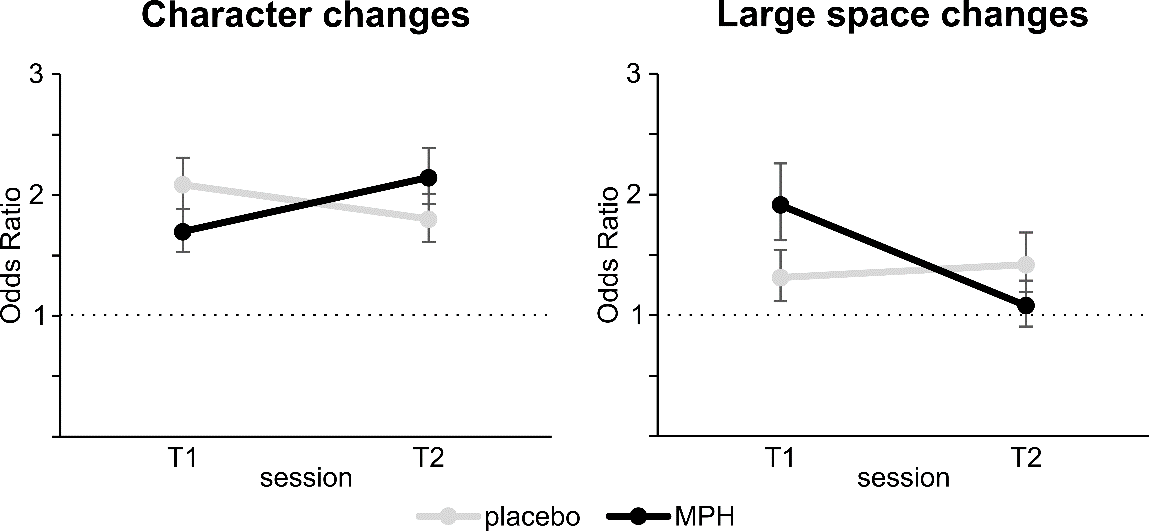


*Supplemental Figure 1.* Interaction of the predictors substance and session on the Character changes (left) and the Large space changes (right). Grey lines indicate placebo, black lines indicate MPH. The values indicate the odds ratios, the error bars indicate the 95% confidence interval. The dotted line indicates an odds ratio of 1, which must not be included in the confidence interval to consider an odds ratio significant.
